# Effects of dichloromethane *Sarcophyton* spp. extract on the lipopolysaccharide-induced expression of nuclear factor-kappa B and inducible nitric oxide synthase in mice

**DOI:** 10.14202/vetworld.2019.1897-1902

**Published:** 2019-12-03

**Authors:** Putut Har Riyadi, Didik Wahyudi, Wendy Alexander Tanod

**Affiliations:** 1 Postgraduate Program, Faculty of Fisheries and Marine Science, Brawijaya University, Malang 65145, East Java, Indonesia; 2 Department of Fisheries Post Harvest Technology, Faculty of Fisheries and Marine Science, Diponegoro University, Semarang 50275, Central Java, Indonesia; 3 Department of Biology, Faculty of Science and Technology, State Islamic University of Maulana Malik Ibrahim Malang, Malang 65144, East Java, Indonesia; 4 Department of Fisheries Product Technology, Institute of Fisheries and Marine (Sekolah Tinggi Perikanan dan Kelautan), Palu 94118, Central Sulawesi, Indonesia

**Keywords:** *Alcyoniidae*, anti-inflammatory, lipopolysaccharide, nitric oxide, soft coral

## Abstract

**Background and Aim:**

The soft coral genus *Sarcophyton* is a source of cembraneterpen. *Sarcophyton* is reported to have anti-inflammatory properties, with the ability to reduce the expression of inducible nitric oxide synthase (iNOS) and inhibit nuclear factor-kappa B (NF-κB) activation. This study aimed to investigate the efficacy of dichloromethane (DCM) extracts of soft coral *Sarcophyton* spp. to inhibit the expression of NF-κB and iNOS induced by lipopolysaccharide (LPS).

**Materials and Methods:**

Crude extracts of *Sarcophyton* spp. were macerated with DCM (1:3 v/v) for 24 h. Thirty-six Balb/c mice were divided into six treatment groups, namely, normal control (without LPS induction), negative control (LPS induction 4 mg/mL), comparative control (LPS+Dexamethasone 6 mg/kg), and 3 concentration groups extract (LPS+50, 125, and 250 mg/kg). The expression of NF-κB and iNOS was measured in each treatment group.

**Results:**

Flow cytometry analysis showed that the relative number of NF-κB^+^ cells increased (18.38±1.24%) in LPS-induced mice compared with normal mice (13.24±1.15%). The *Sarcophyton* spp. DCM extracts decreased the relative number of NF-κB^+^ cells (125 mg/kg: 13.96±0.84%). Immunohistochemical analysis with ImmunoMembrane showed that LPS induction in mice increased iNOS expression when compared to normal mice. The *Sarcophyton* spp. DCM extracts reduced iNOS expression (especially at 125 mg/kg).

**Conclusion:**

DCM extracts of *Sarcophyton* spp. inhibited the activation of NF-κB, resulting in suppressed iNOS expression, which directly inhibits NO production.

## Introduction

Soft coral is a colony of small animals known as polypoid cnidarians (abbreviated as polyps). Soft coral genus *Sarcophyton* belongs to the family Alcyoniidae. *Sarcophyton* is rich in cembraneterpen [1,2]. *Sarcophyton* has a widened shape and mushroom-like form, with sclerite found in the coenenchymal interior tissue [3].

Inducible nitric oxide synthase (iNOS) is present in various types of cells, in response to stimulation by endotoxins and endogenous pro-inflammatory mediators. Stimulation of pro-inflammatory mediators induces iNOS to produce nitric oxide (NO) [4]. Nuclear factor-kappa B (NF-κB) plays an important role in the synthesis of pro-inflammatory cytokines and iNOS expression [5,6]. NF-κB activation increases the expression of pro-inflammatory cytokines, chemokines, and adhesion molecules (ICAM-1, E-selectin, P-selectin, VCAM-1, and HMGB-1) [7,8]. Lipopolysaccharide (LPS) is a glycolipid complex found in the membranes of Gram-negative bacteria and a potent activator of innate immune responses. LPS is the best bacterial immunostimulator to study systemic inflammatory response [9].

Several studies have reported anti-inflammatory properties of *Sarcophyton* through reduced iNOS expression and inhibited NF-κB activation. *Sarcophyton ehrenbergi* and *Sarcophyton crassocaule* have been reported to produce compounds that reduce iNOS expression [10-12]. Furthermore, *Sarcophyton pauciplicatum* inhibits NF-kB activation [13]. The previous studies have also reported the ability of the dichloromethane (DCM) extract of *Sarcophyton* spp. (collected from Palu Bay, Central Sulawesi, Indonesia) to inhibit NO release [14]. In addition, soft coral *Sarcophyton* spp. has the ability to scavenge the free radical DPPH [15].

This study aimed to investigate the ability of DCM extracts from *Sarcophyton* spp. in inhibiting the expression of NF-κB and iNOS induced by LPS in mice.

## Materials and Methods

### Ethical approval

Animal experiments were approved by the Research Ethics Committee, Brawijaya University, Indonesia (Ref. No. 680-Kep-UB).

### Soft coral extraction

Soft coral *Sarcophyton* spp. was obtained from the Palu Bay, Central Sulawesi, Indonesia, at coordinates 43.3 South Latitude and 119.4 East Longitude. Crude extracts were obtained by macerating a wet sample of *Sarcophyton* spp. with DCM: methanol (1:1) (Merck) for 48 h. Subsequently, it was filtered and macerated with DCM (1:3 v/v) for 24 h andthe solvent was evaporated [11].

### Animals and treatment

Experimental animals consisted of 36 male mice (*Mus musculus*) Balb/c strain, obtained from the Integrated Research and Testing Laboratory – Unit IV, Gadjah Mada University, Indonesia. Thirty-six Balb/c mice were divided into six treatment groups of six mice each, namely, normal control (without LPS induction), negative control (LPS induction 4 mg/mL), comparative control (LPS+Dexamethasone 6 mg/kg), extract concentration 1 (LPS+50 mg/kg), extract concentration 2 (LPS+125 mg/kg), and extract concentration 3 (LPS+250 mg/kg). In each treatment group, NF-κB and iNOS expression were observed.

The selected mice were active, with no fur shedding or bent/deformed limbs. Mice were allowed to adapt for 10 days and placed in groups in sterile plastic cages in animal rooms (Department of Biology, FMIPA, Brawijaya University, Indonesia) maintained at a temperature of 24±2°C, with 50-60% humidity and 12 h of dark/light cycles. The mice were fed a standard diet (67.2% carbohydrates, 12.7% protein, and 5.3% fat), which were 10% of the animal body weight and drinking water. Subsequently, mice weighing about 25-30 g were treated with DCM extracts *Sarcophyton* spp. orally for 14 days. On the 14^th^ day before their sacrifice, mice were induced with LPS (LPS *Escherichia coli* O111: B4, List Biological Laboratory, Inc.) by administering as much as 10 µL of 4 mg/ml LPS solution intranasally. In normal controls, an equivalent volume of phosphate-buffered saline (PBS) was administered. After 6-8 h of incubation, the mice were sacrificed by cervical dislocation [16].

### Evaluation of NF-kB expression with flow cytometry

The spleen was collected and washed twice with sterile PBS. It was placed in a tissue culture dish and crushed apart into a single-cell suspension. A single-cell suspension containing 2-3×10^6^ cells was then centrifuged at 2500 rpm (x 700 g) for 5 min at 10°C [17]. The supernatant was discarded and the pellet was stained with FITC-conjugated rat anti-mouse CD11b (Bioss catalog: bs-11127R) against the cell surface marker. It was then incubated in the dark for 20 min at 4°C. The previously stained splenocytes were fixed and then permeabilized using a Cytofix/Cytoperm Kit (BD Biosciences, Pharmingen) according to the manufacturer’s protocol. The supernatant was discarded and the pellet was stained intracellularly with PE/Cy5-conjugated rat anti-mouse NF-κB (Bioss catalog: bs-3543R) followed by incubation in the dark for 20 min at 4°C. The staining combination used for flow cytometric detection was CD11b^+^NF-κB^+^. The final suspension was analyzed using flow cytometry (BD CellQuest program, San Jose, CA).

### Statistical analysis

The data obtained were subjected to analysis by ANOVA (p<0.05); in the cases of any differences between the treatment groups, a subsequent Tukey’s test was performed Statistical Package for Social Science (SPSS) 20.0 for Windows (IBM Corp. NY, USA) was used for data analysis.

### Immunohistochemical examination of iNOS expression

Mice brains were stored in a fixative solution and prepared for immunohistochemistry using a protocol adapted and modified from the previous study [18]. The preparation involved dehydration, clearing, infiltration, paraffin embedding, and sectioning at 4-5 μm. A kit obtained from ScyTek Laboratories was used. The slides were dewaxed, rehydrated, and exposed to xylol and ethanol 3 times for 10 min each. This was followed by peroxidase blocking for 40 min for image analysis with subsequent rinsing in PBS pH 7.5 (3 times). Next, the sections were incubated overnight at 4°C with a primary antibody to NO synthase 2 (Santa Cruz Catalog: SC-7271). The slides were then washed 3 times with PBS pH 7.5 followed by incubation with secondary antibodies with CRF anti-polyvalent biotinylated horseradish peroxidase (HRP) for 1 h. The slides were rinsed 3 times with PBS followed by incubation with UltraTek HRP for 40 min. Thereafter, the slides were rinsed with distilled water until the PBS was removed. After that, the slides were incubated with diaminobenzidine (DAB) chromogen in the DAB substrate (high contrast). The target tissue was examined under a microscope until it turned brown. The substrate reaction was stopped by submerging the slide in distilled water for 5 min. The slides were rinsed with distilled water until the substrate was clean. This was followed by counterstaining with Mayer’s hematoxylin. The counterstain was rinsed with distilled water until clean. When completely dry, the slides were covered with coverslips. Endothelial cells that express iNOS appeared brown under 400× with an Olympus BX-51 microscope in six different fields of view. The images obtained were analyzed using ImmunoMembrane software (URL: http://153.1.200.58:8080/ImmunoMembrane/) and scored as follows: 0/1 for negative/weak expression, 2+ for moderate expression, and 3+ for strong expression [19].

## Results

**Figure-1:**
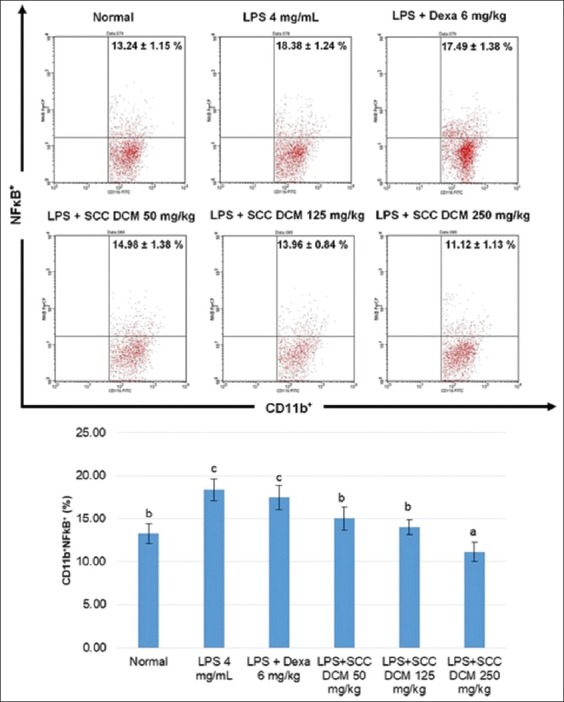
Flow cytometry analysis of nuclear factor-kappa B transcription factors (CD11b^+^NFκB^+^) by *Sarcophyton* spp. dichloromethane extracts. Expressions represented as mean±standard deviation (n=6 for each group). Different letters on the figure considered significantly different for each group at p<0.05.

**Figure-2:**
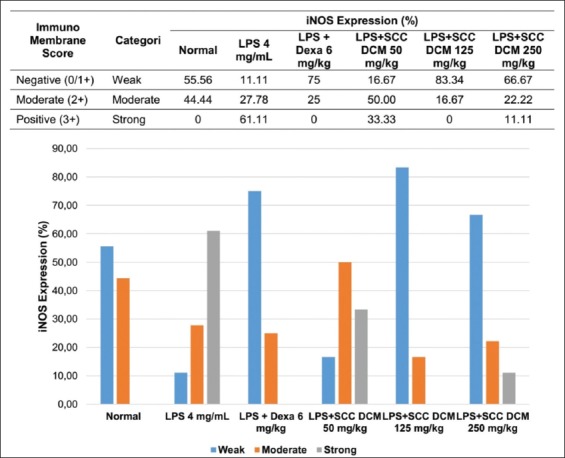
Effects of dichloromethane extract *Sarcophyton* spp. on inducible nitric oxide synthase expression with ImmunoMembrane analysis.

Flow cytometry analysis showed that the relative number of NF-κB^+^ cells was higher in LPS-induced mice compared with normal mice (p<0.05). The DCM extracts of *Sarcophyton* spp. reduced the relative number of NF-κB^+^ cells (p<0.05) (Figures-1 and Figure-2). Although dexamethasone also showed a decrease, it was not statistically significant compared with the mice subjected to induction with 4 mg/mL LPS.

**Figure-3:**
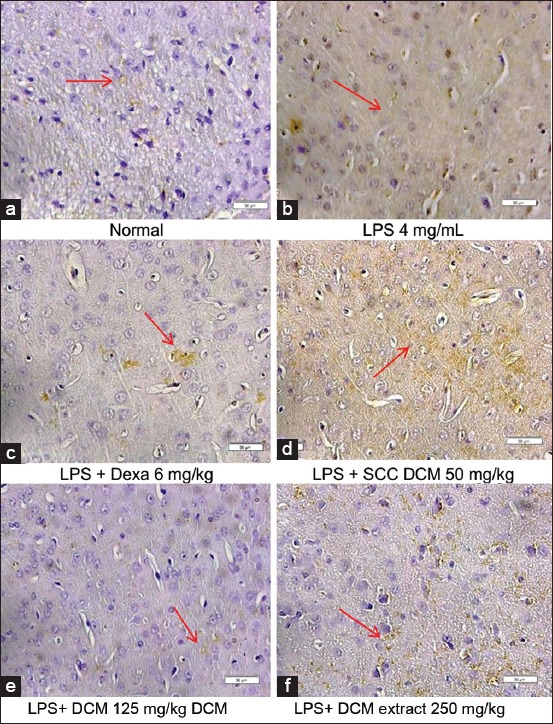
Immunohistochemistry examination showed inducible nitric oxide synthase expression in lipopolysaccharide (LPS)-induced mice. (a) Normal; (b) LPS 4 mg/mL; (c) LPS+ 6 mg/kg dexamethasone; (d) LPS+50 mg/kg dichloromethane (DCM) extract *Sarcophyton* spp.; (e) LPS+125 mg/kg DCM extract *Sarcophyton* spp.; (f) LPS+250 mg/kg DCM extract *Sarcophyton* spp. 400×; 1 bar=50 µm; location of observation: Cerebral cortex; location of expression: Endothelial cells around blood vessels; brown color showed positive reaction.

ImmunoMembrane analysis showed that LPS induction in mice increased iNOS expression compared to normal mice (Figure-2). Treatment with *Sarcophyton* spp. DCM extracts and dexamethasone reduced iNOS expression (Figure-3). Although iNOS expression increased again in the 250 mg/kg treatment group, it did not exceed the expression in LPS-induced mice.

## Discussion

LPS is an endotoxin found in Gram-negative bacteria that play a role in forming cell walls and acts as a virulence factor [20-22]. LPS is an innate immune response element and is the sensor with the best characteristics for TLR4-mediated inflammatory response and other important events needed to initiate the innate immune response [23]. LPS stimulation affects the synthesis of interleukin (IL)-1β, tumor necrosis factor-α, IL-6, and NF-κB ligand receptors [24,25]. The transcription factor NF-κB binds to inflammatory mediator protein promoters resulting in the production and secretion of pro-inflammatory cytokines [26,27]. The previous study showed that DCM extracts of *Sarcophyton* spp. inhibited NO production [14]. This inhibition of NO production was presumably achieved by regulating the level of iNOS and its upstream molecules in LPS-induced macrophage cells [28]. The transcription factor NF-κB promotes the expression of iNOS when stimulated by LPS [29]. The results of this study showed that the DCM extracts of *Sarcophyton* spp. inhibited the activation of NF-kB by decreasing its transcriptional activity and nuclear translocation in LPS-induced macrophage cells.

NF-κB reduction occurs through the expression of IκBα suppressor, which inhibits and reduces the production of pro-inflammatory cytokines. The *Sarcophyton* spp. DCM extracts showed the ability to reduce the relative number of NF-κB^+^ cells. It is presumably because the extract can maintain the integrity of IκBα so that it can inhibit the translocation of NF-κB toward the nucleus of macrophage cells. LPS induction in this study caused a significant increase in NF-κB activation when compared to normal controls (without LPS induction). This trend is consistent with the theory that LPS induction stimulates NF-κB activation and expression of pro-inflammatory molecules [5].

iNOS, as one of the three key enzymes that catalyze the production of NO, is an important regulatory molecule in inflammatory response and cancer development [30]. iNOS can be induced after activation by various stimuli, especially pro-inflammatory mediators [31]. The NF-κB signaling pathway plays an important role in regulating the inflammatory response through the transcription of iNOS genes and cytokines [32]. Cells exposed to external pro-inflammatory stimuli such as mitogens, pro-inflammatory cytokines, and LPS cause rapid phosphorylation of IκB by IκB kinase, resulting in nuclear translocation of NF-κB [33]. In the nucleus, NF-κB induces the transcription of many pro-inflammatory gene targets, including iNOS. iNOS then facilitates the conversion of L-arginine to L-citrulline and large amounts of NO [32]. iNOS gene expression is regulated at the transcriptional level. iNOS induction is primarily dependent on the activity of the transcription factors that interact with cognate cis-acting elements in the iNOS promoter [34-37].

In this study, DCM extracts of *Sarcophyton* spp. decreased the expression of NF-κB. It was suggested that the suppression of iNOS expression is related to the deactivation of the NF-κB pathway. The results of this study provided a molecular basis for the anti-inflammatory response of *Sarcophyton* spp. DCM extracts. These extracts have the potential to be used for the discovery/development of pharmaceutical agents for various diseases with an excessive inflammatory response.

## Conclusion

From the results of this study, it can be concluded that the DCM extract of *Sarcophyton* spp. inhibited the activation of NF-kB, thereby leading to suppress iNOS expression that is directly related to the inhibition of NO production. It can prevent the excessive inflammatory response triggered by LPS induction.

## Authors’ Contributions

PHR contributed to the conception, research design, data acquisition, and designed the manuscript. DW did analysis and/or interpretation of data. WAT critically revised and improved the manuscript. All authors read and approved the final manuscript.
